# Identification of a novel peptide targeting TIGIT to evaluate immunomodulation of ^125^I seed brachytherapy in HCC by near-infrared fluorescence

**DOI:** 10.3389/fonc.2023.1143266

**Published:** 2023-04-14

**Authors:** Peng Zeng, Duo Shen, Wenbin Shu, Shudan Min, Min Shu, Xijuan Yao, Yong Wang, Rong Chen

**Affiliations:** ^1^ Department of Oncology, Zhongda Hospital, Medical School, Southeast University, Nanjing, Jiangsu, China; ^2^ Department of Gastroenterology, The Second People’s Hospital of Changzhou, Nanjing Medical University, Changzhou, Jiangsu, China; ^3^ Department of Gastrointestinal Surgery, The Second Affiliated Hospital of Nanchang University, Nanchang, Jiangxi, China; ^4^ Jiangsu Key Laboratory of Molecular and Functional Imaging, Department of Radiology, Zhongda Hospital, Medical School, Southeast University, Nanjing, Jiangsu, China; ^5^ Center of Interventional Radiology and Vascular Surgery, Department of Radiology, Zhongda Hospital, Medical School, Southeast University, Nanjing, Jiangsu, China

**Keywords:** hepatocellular carcinoma, TIGIT, ^125^I seed brachytherapy, peptide, NIRF

## Abstract

**Introduction:**

Hepatocellular carcinoma (HCC) has very poor prognosis due to its immunosuppressive properties. An effective measure to regulate tumor immunity is brachytherapy, which uses ^125^I seeds planted into tumor. T cell immune receptors with immunoglobulin and ITIM domains (TIGIT) is highly expressed in HCC. The TIGIT-targeted probe is expected to be an effective tool for indicating immunomodulation of ^125^I seed brachytherapy in HCC. In this study, We constructed a novel peptide targeting TIGIT to evaluate the immune regulation of ^125^I seed brachytherapy for HCC by near-infrared fluorescence (NIRF).

**Methods:**

Expression of TIGIT by immunofluorescence (IF) and flow cytometry (FCM) in different part and different differentiated human liver cancer tissues was verified. An optical fluorescence probe (Po-12) containing a NIRF dye and TIGIT peptide was synthesized for evaluating the modulatory effect of ^125^I seed brachytherapy. Lymphocytes uptake by Po-12 were detected by FCM and confocal microscopy. The distribution and accumulation of Po-12 in vivo were explored by NIRF imaging in subcutaneous and orthotopic tumors. IHC and IF staining were used to verify the expression of TIGIT in the tumors.

**Results:**

TIGIT was highly expressed in HCC and increased with tumor differentiation. The dye-labeled peptide (Po-12) retained a stable binding affinity for the TIGIT protein *in vitro*. Accumulation of fluorescence intensity (FI) increased with time extended in subcutaneous H22 tumors, and the optimal point is 1 h. TIGIT was highly expressed on lymphocytes infiltrated in tumors and could be suppressed by ^125^I seed brachytherapy. Accumulation of Po-12-Cy5 was increased in tumor-bearing groups while declined in 125I radiation group.

## 1 Introduction

Hepatocellular carcinoma (HCC) is the fifth most common cancer and the second leading cause of cancer-related death worldwide (1). The treatment of advanced HCC has been a dilemma because of its self-immune tolerance (2). Local radiotherapy (RT) is an effective immunomodulatory measure for tumors (3). ^125^I seed implantation brachytherapy is a new type of RT that has been widely used in the treatment of a variety of tumors, including liver cancer (4–8). ^125^I seed is a kind of single miniature radioactive source with low dose rate. The core of this seed is palladium wire of ^125^I radioactive nuclide, encased in cylindrical sealed titanium alloy tube, with half-life of 59.43 days, average energy of 27 ~ 35 keV and radiation distance of 1.7 ~ 2.0 cm.^125^I seed brachytherapy has a good safety profile for the treatment of HCC (9). Increasing evidence has confirmed that ^125^I seed brachytherapy inhibits tumor growth and activates antitumor immunity (10–13). However, RT alone is not enough to prevent tumor recurrence and metastasis. Combined RT can further promote this immunodulatory effect, in which the combination of nano-materials such as photothermal therapy (PTT) and photodynamics therapy (PDT) have a significant effect (14–16). More important, RT can also result in immunosuppression with the.accumulation of radiation dose (17). Nevertheless, there is currently no accurate method for evaluating immune molecule changes in the tumor microenvironment (TME) for clinical treatment. Therefore, real-time and dynamic monitoring of these molecules is needed to detect changes in immune translation and provide guidance for immunotherapy.

T cell immune receptor with immunoglobulin and ITIM domains (TIGIT) is a receptor of the Ig superfamily. It plays a key role in limiting adaptive and innate immunity and is involved in tumor immune surveillance, mainly expressed on T cells, natural killer cells (NK), and other antigen-presenting cells (APCs), which can reduce cytokine production and show strong immunosuppressive effects (18). Considering that NK cells account for a large proportion in liver, and TIGIT is expressed on both NK and T cells, TIGIT has been reported to be an important inhibitory immune checkpoint (ICP) in HCC (19). RT regulates the expression of TIGIT in tumors. RT combined with anti-TIGIT is a good anti-tumor strategy (20, 21). Consequently, TIGIT can be used as an indicator of tumor immunoactivity. Hence, we used TIGIT as a marker to reflect the immunoregulation of ^125^I seed brachytherapy in HCC.

The development of molecular imaging technology provides the possibility for the dynamic assessment of tumor immune microenvironment (TIME) changes in real time. It is reasonable to measure immunoactivities by molecular imaging of predictive biomarkers in tumors. Near-infrared fluorescence (NIRF) emitters have been widely used in the real-time imaging of tumors because of their excellent tissue penetration and target-background contrast (22, 23). Therapeutic targeted molecules and immune checkpoints (ICPs) labeled with NIRF dye have been tested in the evaluation of cancer therapy, demonstrating ideal safety and high accuracy in identifying the TME (24–26).

NIR imaging probes are usually composed of NIR dyes and targeting groups (including antibodies and their fragments, peptides, small molecules, etc.), which can bind to specific molecules in the process of tumorigenesis and development to achieve dynamic tracing of the TME (27–29). Among these, peptides stand out among many targeting groups because of their low immunogenicity, strong tissue penetration, fast blood clearance, and relatively simple production process (30, 31). Phage display technology combines the antigen recognition ability of recombinant proteins and is an efficient screening system to generate peptides against specific molecules or tumor structures. Therefore, it has great prospects in the development of tumor-specific peptides (32).

**Figure 1:**
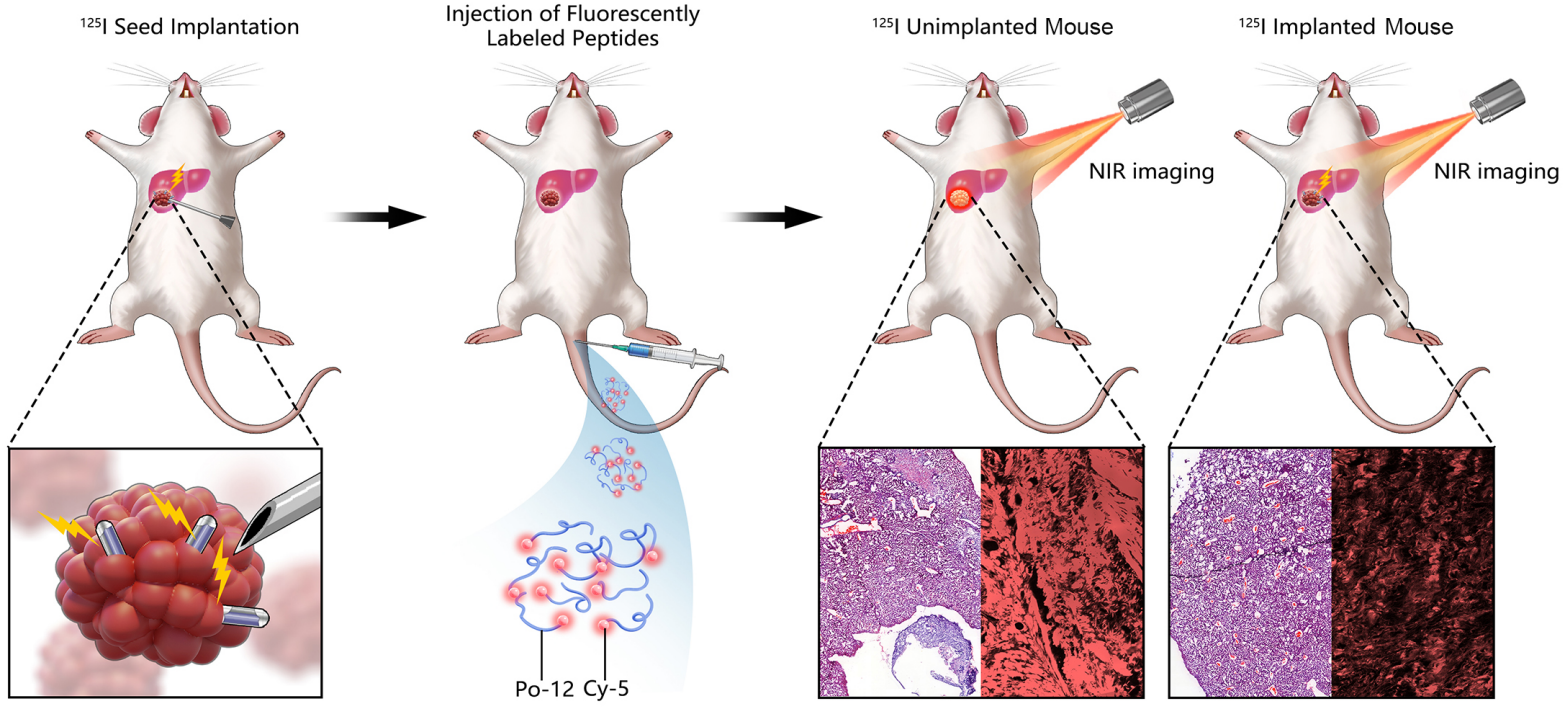
Diagram showing the general scheme for using the TIGIT probe in near-infrared fluorescence (NIRF)-guided ^125^I seed brachytherapy in orthotopic HCC. ^125^I seed was used to implanted into orthotopic tumor for radiation. Po-12 conjugated Cy5 targeting TIGIT was administrated by tail vein. Change of TIGIT expression was detected by NIR imaging.

As TIGIT is highly expressed in HCC, and is a new immunotherapeutic target that may be regulated by RT (33), we introduced ^125^I seed implantation into the tumor for brachytherapy and regulated the expression of TIGIT. We also designed a 12-amino acid peptide targeting TIGIT to bind to lymphocytes in HCC. This peptide was combined with Cy5 to further evaluate the targeting efficacy of the probe in HCC before and after radiotherapy under NIRF, which can indicate the degree of immune regulation of HCC by ^125^I seed brachytherapy (**Figure 1**).

## 2 Methods

### 2.1 Expression and purification of recombinant TIGIT antigen

In order to obtain the recombinant TIGIT protein, the TIGIT gene (available in PubMed) was cloned into the BamHI and EcoRI sites of PET28-A vector (+) and transformed into BL21(DE3) competent Escherichia coli cells, which were cultured in Luria broth at 37 ^°^C, containing ampicillin (OD values of 0.6-0.8). Subsequently, 1μM isopropyl-β-D-thio-galactoside (IPTG) was added to the culture to induce protein expression. Bacterial cultures were harvested and centrifuged at 5000 rpm for 10 min, after which the precipitate was resuspended in lysis buffer containing 8 M urea and 50 mM Tris (pH 7.4). After complete decomposition at high pressure, lysed bacteria were centrifuged at 15000 rpm for 30 min and loaded onto a nickel resin-bound (column affinity chromatography) column. The recombinant TIGIT protein was eluted with a highly stringent buffer containing 300 mM imidazole and verified by 10% SDS-PAGE and Coomassie Brilliant Blue staining to obtain purified recombinant TIGIT protein.

### 2.2 Screening of TIGIT targeted peptides

The resulting purified TIGIT protein was coated onto a 96-well plate and used for subsequent peptide screening. Phage display technology was used for the screening. Fifty microliters of phage supernatant were added to a 96-well plate coated with TIGIT protein for screening. 0.2 M Gly-HCl PH2.2 was used for elution and 1M Tris-HCl PH9.1 was used for neutralization. The neutralization solution (containing bacteriophages) was diluted 1000 or 10000 times and then added to E. coli for amplification. After three rounds of screening, the desired affinity clone target was obtained. The cloned target was sequenced using DNA and the target amino acid sequence was obtained by reverse sequencing. A sequence with a high affinity and the highest occurrence times was selected for chemical synthesis. To better link the Cy5 fluorophore, the Cy5 fluorophore was first added to the synthesized amino acid sequence and then the Cy5 fluorophore was added to the N-terminus of the amino acid sequence. All peptides were chemically synthesized using the solid-phase Fmoc method and purified by high-performance liquid chromatography (HPLC) and electrospray ionization mass spectrometry to a minimum purity of 95%.

### 2.3 Cell culture and animal models

H22 cells (Chinese National Collection of Authenticated Cell Cultures) were cultured in Roswell Park Memorial Institute 1640 medium (RPMI 1640, Gibco, USA), containing 10% fetal bovine serum (FBS, Gibco, USA) and 10% penicillin/streptomycin (P/S, Gibco, USA) at 37° in a humidified atmosphere containing 5% CO2. All animal experiments were approved by the Animal Ethics Committee of Southeast University and conducted in compliance with the Regulations for the Administration of Affairs Concerning Experimental Animals of China. Six-week-old male BALB/c mice (Vital River Laboratory Animal Technology, China) were housed at the Animal Center of the Southeast University laboratory. 1×10^6^ H22 cells were used to induce subcutaneous tumors by an injection into the back of each mouse and tumor tissue was used to generate orthotopic hepatic tumors by implantation into the liver. The mice were anesthetized with an intraperitoneal injection of 60 mg/kg sodium pentobarbital. ^125^I seeds (activity of 0.8 mCi) were implanted into the tumor for radiation.

### 2.4 Cell sorting of T lymphocytes

The tumor tissues of each group were minced into small fragments and digested with tissue digestive enzymes(Miltenyi Biotec, Germany)at 37 °C for 40 min. Single cells were collected by filtration through a 70 μm colander (BD Biosciences, USA). T lymphocytes were sorted using magnetic beads and a CD45^+^ lymphocyte isolation kit (STEMCELL Technologies, Canada).

### 2.5 Flow cytometry

Isolated CD45^+^ lymphocytes were collected at 1 × 10^6^ cells/sample and incubated with 300 μL control Con-12 or Po-12 (10 μg/mL) at 4 °C for 15 min. After incubation, the cells were washed with PBS 3 times and then resuspended in 400 μL staining buffer. Fluorescence analysis was performed using a flow cytometer (BD Biosciences) with a count of 1×10^6^ living cells per sample. The results were analyzed by flow cytometry using Flow Jo software for 3 times (v7.6, OR, USA).

### 2.6 Immunofluorescence staining

Isolated CD45^+^ lymphocytes were seeded in a confocal chamber at 1 × 10^6^ cells/well for 24 h and fixed with 4% paraformaldehyde at room temperature for 20 min. The cells were incubated with Po-12-Cy5 or Con-12-Cy5 at 4°C overnight. After staining with 4’ 6-diamidino-2-phenylindole (DAPI), cells were imaged using a confocal microscope (FV3000; Olympus, Japan). The prepared tumor sections were also subjected to immunofluorescence (IF), and the tumor tissues were resected and frozen for IF imaging. Slides were stained with DAPI and analyzed using a confocal microscope (FV3000, Olympus, Japan).

### 2.7 Western blotting

Isolated CD45^+^ lymphocytes were lysed to concentrate proteins using RIPA lysis buffer (Beyotime Biotechnology, China). Cell extracts were clarified by centrifugation, and protein concentrations were determined using the BCA assay. Protein extracts were separated by SDS-PAGE, transferred to microporous polyvinylidene difluoride membranes (Roche, USA), and blocked using 5% BSA. Then the membranes were incubated with anti-TIGIT polyclonal antibody (Abbexa, UK) or GAPDH monoclonal antibody (Cell Signaling Technology, MA, USA) at 4°C overnight. After washing, the membranes were incubated with HRP-conjugated secondary antibodies (Cell Signaling Technology, USA) at room temperature for 1 h. Protein bands were detected with enhanced chemiluminescence (ECL) and imaged using a chemiluminescence system (Bio-Rad, USA). The above experimental procedures were repeated 3 times.

### 2.8 Near-infrared fluorescence imaging

Six H22 tumor-bearing mice were randomly divided into two groups and intravenously injected with 20 μg Po-12-Cy5-peptide or Con-12-Cy5-peptide. After anesthesia with isoflurane in oxygen, *in vivo* fluorescence imaging was performed using an IVIS-Spectrum system (Perkin Elmer, Santa Clara, CA, USA) at several time points (0.5, 0.75, 1, 2, 4, and 8 h). The excitation and emission wavelengths of the probe were 620 and 670 nm, respectively. The mice were sacrificed after injection of the peptide; their tumors and major organs were dissected for ex vivo NIR imaging.

### 2.9 Statistical analysis

All data are presented as mean ± standard deviation (SD). Statistical significance between groups was determined using two-tailed Student’s t-test or one-way analysis of variance (ANOVA). The threshold of statistical significance was set at *P* < 0.05 (*P < 0.05, **P < 0.01). Statistical analyses were performed using the GraphPad Prism software (V9.0, CA, USA).

## 3 Results

### 3.1 TIGIT expression in human HCC samples

**Figure 2:**
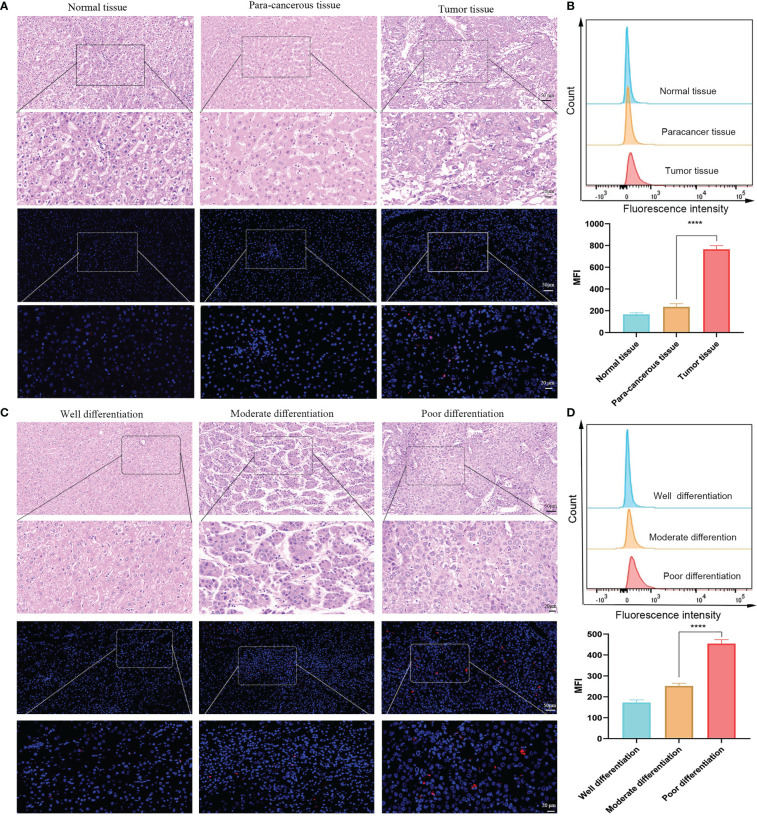
TIGIT expression in human HCC. **(A)** Representative IF staining of TIGIT in different parts of tissue. **(B)** Flow cytometric analysis of TIGIT expression on lymphocytes in different parts of tissue (n=3). **(C)** Representative IF staining of TIGIT in various differentiated tumor tissue. **(D)** Flow cytometric analysis of TIGIT expression on lymphocytes in different differentiated tumor tissue (n=3). (Scale bar: Up, 50 μm; Down, 20 μm). ****P < 0.01.

In experiments examining the expression of TIGIT protein in human HCC, different parts of the tissue (normal, paracancer, and tumor tissue) and differentiated tumor tissues (well, moderate, and poorly differentiated tumors) were collected. H&E and IF staining was used to assess TIGIT expression in each group. The probe distribution assay of IF showed extensive accumulation of fluorescence in tumor tissue compared to paracancerous tissue but was negligible in normal tissue (**Figure 2A**). Furthermore, fluorescence intensity (FI) increased with the degree of malignancy, which revealed a stronger accumulation in poorly differentiated tumors than in moderately differentiated tumors, while the well-differentiated tumor displayed the least FI (**Figure 2C**). To further verify the expression of TIGIT, we detected the expression of TIGIT on the surface of lymphocytes in each tissue using flow cytometry (FCM), and the results showed a consistent trend (**Figures 2B–D**, *P* < 0.01). These results indicate that TIGIT was highly expressed in HCC and increased with tumor differentiation.

### 3.2 Identification and synthesis of TIGIT-targeted peptides

**Figure 3:**
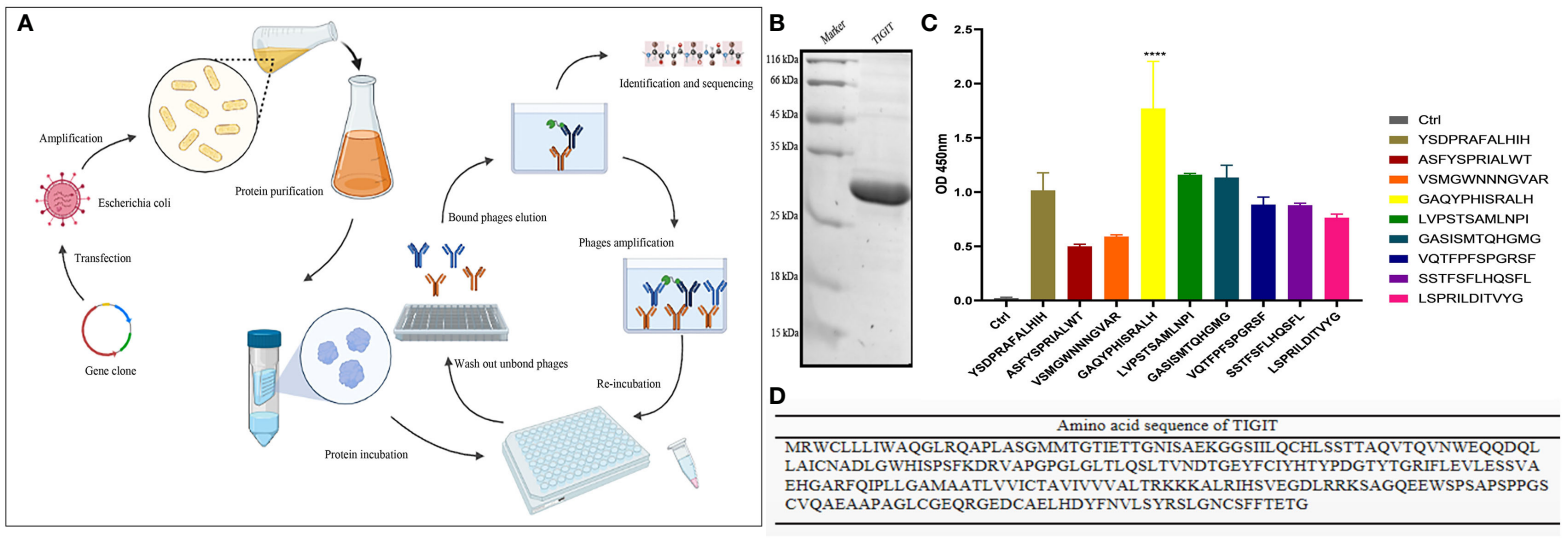
Identification of TIGIT-targeted peptides. **(A)** A flow chart for screening of TIGIT-targeted peptide. **(B)** Amino acid sequence of TIGIT protein. **(C)** Coomassie Brilliant Blue staining of TIGIT protein after purification. **(D)** The binding affinities of selected nine peptide. The OD values were analyzed by phage ELISA. ****P < 0.01.

TIGIT protein was successfully purified. Three rounds of incubation and screening of expressed proteins were performed (**Figure 3A**). Significant enrichment of recovered phages was observed (**Figure 3B**). A consistently predicted molecular weight of approximately 29 kDa was determined by Coomassie Brilliant Blue staining (**Figure 3C**). After the last round of screening, 19 clones were randomly selected, verified by enzyme-linked immunosorbent assay (ELISA), and sequenced by high-throughput sequencing. The absorbance of the two highly repetitive peptide sequences at 450 nm was significantly higher than that of the control sample (**Figure 3D**). The high frequency of the peptide sequences indicated efficient enrichment during the screening process. The peptide sequence GAQYPHISRALH (named Po-12), with an OD equal to 10 times that of the control, was selected as the best candidate peptide for subsequent studies, and the peptide with sequence shuffling (named Con-12) was used as the control peptide (**Figure 2D**). The molecular structure of Po-12-Cy5 is presented in **Figure S1A**. Cy5 fluorophore was added to (red marker) the N-terminus of the naked peptide (**Figure S1A**). The mass to charge ratio (M/Z) of Po-12-Cy5 by mass spectrometry was determined at 2231.58(**Figure S1B**), and the retention time of peptide purification by HPLC was 11.023 min (**Figure S1C**).

### 3.3 Binding of TIGIT-targeted peptide to lymphocytes

**Figure 4:**
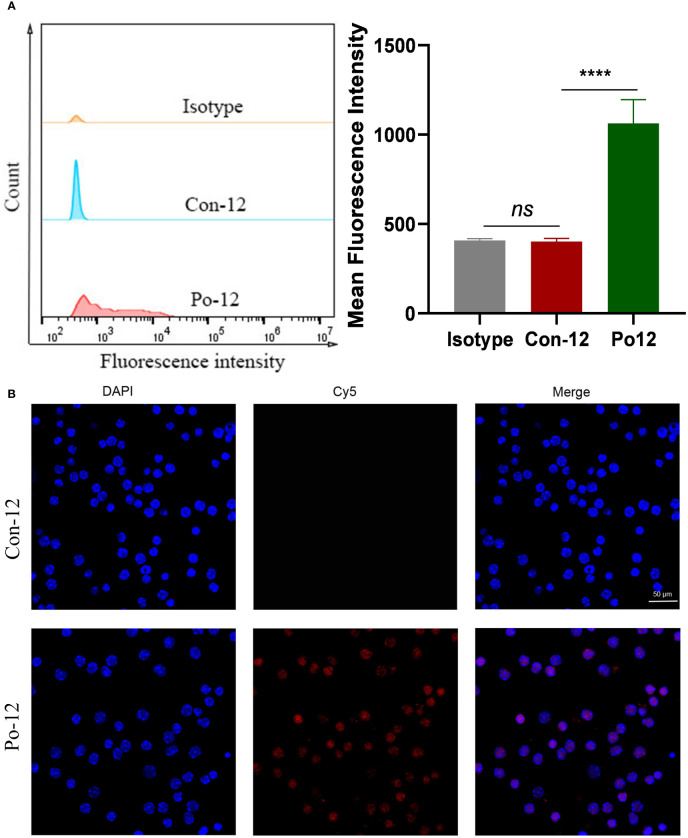
Binding of TIGIT-targeted peptide to lymphocytes. **(A)** FCM analysis of lymphocytes after incubation with Con-12-Cy5 or Po-12-Cy5 peptide (n=3). **(B)** Confocal images of lymphocytes after treatment with Con-12-Cy5 or Po12-Cy5 peptide. Scale bar: 50 μm. ns, no significance; ****P < 0.01.

The *in vitro* specificity of Po-12 to the TIGIT protein was evaluated using FCM. The results revealed that lymphocytes in the Po-12 group showed a stronger absorption of fluorescence intensity than those in the Con-12 group and isotype group (**Figure 4A**, *P* < 0.01). Confocal microscopy imaging was used to evaluate the cellular binding of the probes. Strong membranous binding was observed in lymphocytes treated with Po-12-Cy5, whereas almost no fluorescence was found in Con-12-Cy5-treated one (**Figure 4B**). These data indicated that the dye-labeled peptide retained a stable binding affinity for the TIGIT protein *in vitro*.

### 3.4 NIRF imaging of tumors models

**Figure 5:**
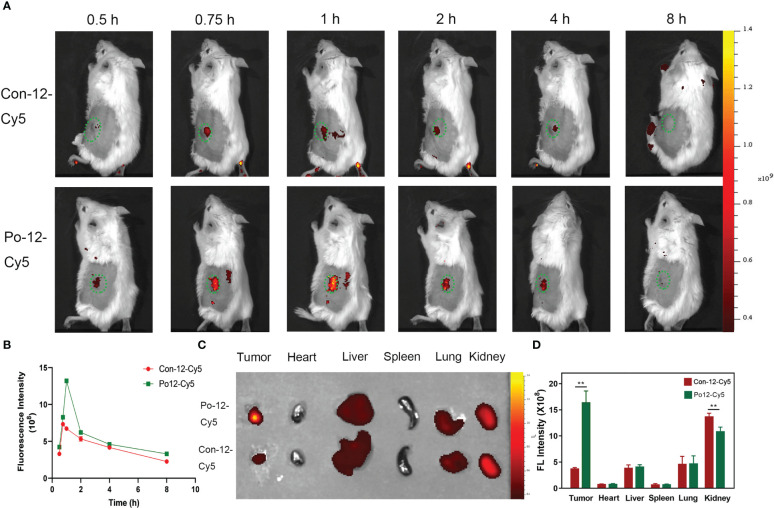
*In vivo* imaging of H22 subcutaneous tumors and biodistribution of the probe. **(A)** *In vivo* imaging post-injection of probes and **(B)** quantification of fluorescence intensity (n=3). **(C)** *Ex vivo* imaging of tumor and normal organs (Tumor, heart, liver, spleen, lung, kidney) and **(D)** quantification of fluorescence intensity (n=3). **P < 0.01.

NIRF imaging was performed in tumor-bearing BALB/c mice by intravenous injection of Cy5-peptides. Accumulation of FI increased in subcutaneous H22 tumors from 0.5 h until 1 h, after which the FI began declining. Quantitative analysis showed that the mean fluorescence intensity (MFI) of Po-12-Cy5 was significantly higher than that of Con-12-Cy5 (**Figures 5A, B**, P < 0.01). Ex vivo optical imaging of the tumors and main organs was performed 1 h post-injection. The quantification of FI corroborated the visualization of *in vivo* optical imaging. Biodistribution analysis indicated that Po-12-Cy5 showed prominent renal clearance. The enrichment of Po-12-Cy5 in H22 tumors was the highest in all organs except the heart (**Figures 5C, D**).

### 3.5 TIGIT expression and cellular uptake of probes in lymphocytes after brachytherapy

**Figure 6:**
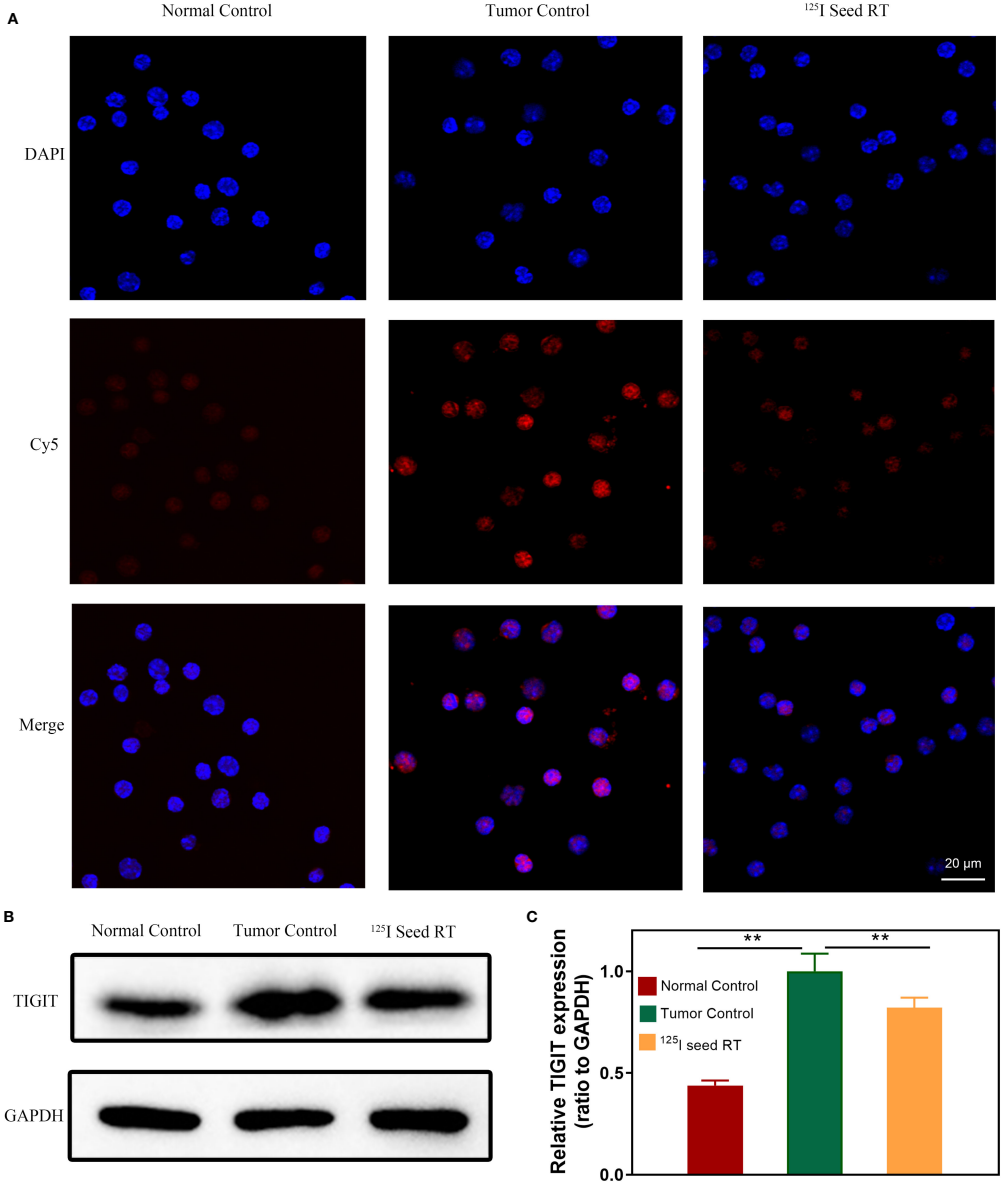
TIGIT expression and cellular uptake of probes in lymphocytes. **(A)** Confocal microscopic imaging of the cellular binding of probes in lymphocytes. **(B)** Western blotting of TIGIT protein in lymphocytes and **(C)** quantification of TIGIT expression (n=3). Scale bar: 20 μm. **P < 0.01.

To detect the effect of brachytherapy on the expression of TIGIT in HCC, we established mouse subcutaneous tumor models under ^125^I seed radiation. Lymphocytes from each group were isolated from tumor tissue using magnetic beads and incubated with Po12-Cy5. Confocal microscopy imaging was also used to evaluate the cellular uptake of the TIGIT-targeted probe in lymphocytes after radiation. The results showed that the expression of TIGIT in the tumor control group was significantly increased compared with that in the normal control group and decreased in the ^125^I seed radiation group (**Figure 6A**). Consistent with confocal microscopy images, TIGIT protein expression in each group extracted from isolated lymphocytes assessed by western blotting also exhibited a similar tendency (**Figures 6B, C**, *P* < 0.01). These results demonstrated that TIGIT was highly expressed on lymphocytes infiltrated in tumors and could be suppressed by ^125^I seed brachytherapy, which suggested that this probe may not only visualize the expression changes of TIGIT in tumors but also provide dynamic guidance for RT in TME regulation.

### 3.6 NIRF-guided TIGIT expression in tumor model of HCC after brachytherapy

**Figure 7:**
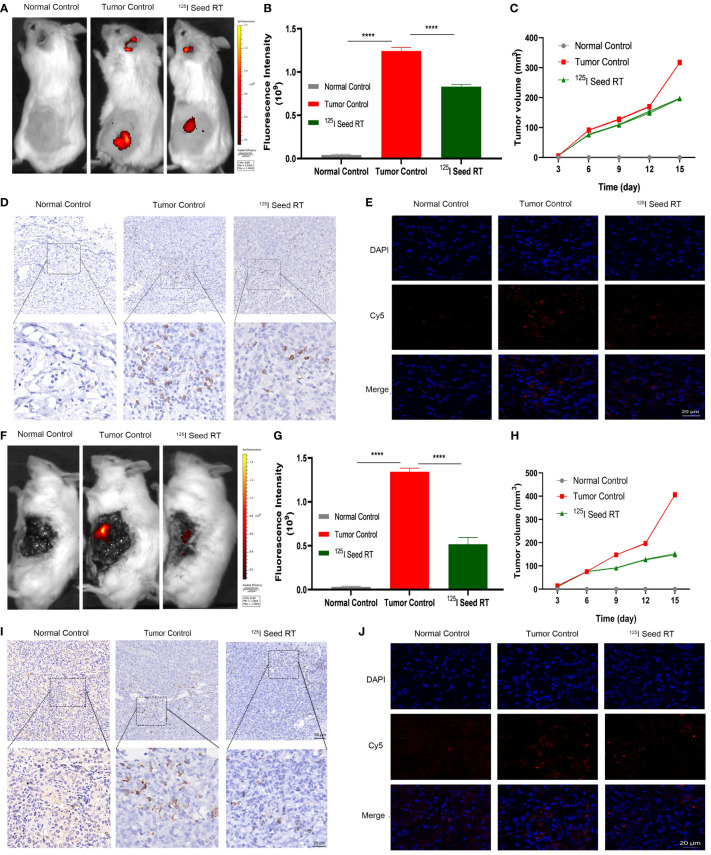
Fluorescence imaging in subcutaneous and orthotopic model of HCC. **(A–F)** Imaging of subcutaneous and orthotopic tumor after infusion of probes. **(B–G)** Quantification of FI in subcutaneous and orthotopic tumor (n=3). **(D–I)** IHC of TIGIT expression in subcutaneous and orthotopic tumor. **(E–J)** Probe distribution evaluated *via* IF staining of frozen sections from subcutaneous and orthotopic tumor. **(C–H)** Tumor volume of subcutaneous and orthotopic tumor after ^125^I seed radiation (n=4). Scale bar: 50 μm. Scale bar: 20 μm. ****P < 0.01.

To verify the effect of radiation on TIGIT expression and the targeting of the probe in HCC, subcutaneous and orthotopic HCC tumor models were established in mice. The probe was injected into the tail vein, and accumulation of Po-12-Cy5 was increased in both subcutaneous and orthotopic tumors, while the normal control group without tumor showed no change. Quantification analysis revealed that FI in ^125^I radiation group declined significantly than tumor control one (**Figures 7A–G**, *P* < 0.01). The results of tumor growth curve were also consistent with IF, and the growth of subcutaneous and orthotopic tumor was inhibited observably (**Figures 7C–H**, *P* < 0.01). In addition, the tumors were excised for IHC and IF staining. Results revealed that TIGIT protein expression in IHC was negligible in the normal control group, while it was highly expressed in the tumor-bearing group and downregulated in the ^125^I radiation group (**Figures 7D–I**). In agreement with IHC, extensive accumulation of Po-12-Cy5 was observed in tumor-bearing groups compared to that in the normal control group, and also declined in the ^125^I radiation group (**Figures 7E–J**). These results reveal that Po-12-Cy5 has excellent TIGIT-positive tumor-targeting potential and can be used as a significant indicator of radiation immune regulation.

## 4 Discussion

Evaluation of the immune response has always been a challenge in tumor therapy because of the potential reversion or pseudoprogression (34). However, there is still a lack of effective non-invasive real-time dynamic evaluation measures based on immune molecules. The advent of molecular imaging technology provides an opportunity for noninvasive observation of abnormal immune molecular events *in vivo*. In response to the expression of TIGIT protein in cancer, a variety of therapeutic antibodies have been developed in phase I-II clinical trials (35, 36). Probe navigation systems for recognizing tumor molecules mainly include antibodies, peptides, and small molecules (27–29). Among them, peptides have been valued for their ability to bind hidden epitopes because of their smaller molecular weight (31). With the emergence and rapid development of phage display technology, new peptidyl molecular probes have greatly promoted the detection of tumor molecules, showing great potential for clinical exploration (37, 38).

To the best of our knowledge, the indication of ICP by optical labeling of peptide targeting TIGIT under real-time NIF for RT regulation has not been reported. In this study, the target peptide of TIGIT was identified using phage display technology. To determine the expression of TIGIT in HCC, we first collected different parts and differentiated human HCC tissues and detected the expression of TIGIT by IF staining and FCM. The results showed that the expression of TIGIT in tumor tissues was significantly higher than that in adjacent and normal tissues, and the expression of TIGIT in poorly differentiated liver cancer tissues was significantly higher than that in moderately and well-differentiated tissues. Furthermore, we isolated lymphocytes from mouse tumors and verified the high binding affinity of the Cy5-conjugated peptide (Po-12-Cy5) to lymphocytes by FCM and immunofluorescence confocal assay *in vitro*. *In vivo* optical imaging further verified the targeting ability of Po-12-Cy5 in a subcutaneous HCC model. The results showed that the fluorescence uptake of Po-12-Cy5 was significantly stronger than that of Con-12-Cy5, peaking rapidly within 1 h, and gradually declining over 7 h. In addition, the biodistribution results showed that the fluorescence intensity in the tumor was significantly higher than that of the hybrid peptide, which was consistent with *in vivo* observations. In addition, the uptake of Po-12-Cy5 in the kidney was higher than that in other organs, indicating that the kidney may be the main excretion route. This is consistent with what has been reported in a series of other literatures (39–41). These results indicate that Po-12-Cy5 has a good targeting effect on TIGIT in HCC and can be used as an important indicator of changes in the immune microenvironment in HCC. Therefore, we planned to use it to dynamically monitor the regulation of TIGIT by ^125^I seed RT in real-time.

Based on the specificity of the Po-12-Cy5 probe for TIGIT in liver cancer, we constructed a tumor brachytherapy model using ^125^I seed implantation and isolated tumor-infiltrating lymphocytes and compared the expression of TIGIT under ^125^I seed RT, tumor control, and normal control groups *in vitro*. Immunofluorescence confocal analysis showed that the fluorescence uptake in the tumor group was significantly higher than that in the normal control group, while that in the ^125^I seed RT group was downregulated. The expression of the TIGIT protein in each group also demonstrated this trend. To further demonstrate the indication of the Po-12-Cy5 probe on TIGIT by RT, we constructed subcutaneous and orthotopic tumor models of HCC in mice and further evaluated the targeting of the Po-12-Cy5 probe *in vivo*. Quantitative analysis showed that the FI of the ^125^I seed RT group was significantly lower than that of the tumor control group in both the subcutaneous and orthotopic tumor models. IF and IHC staining of tumor tissues also showed this trend. These results indicate that the Po-12-Cy5 probe has precise targeting of TIGIT in HCC and can be used as an indicator of RT immunoregulation, which has important clinical significance for guiding HCC immunotherapy.

However, there are still some problems associated with the clinical translation of the Po-12-Cy5 probe. First, the clinical safety of the NIF dye-Cy5, was not confirmed. Nonetheless, NIR-II dye-indocyanine green (ICG) has been approved for clinical use by the Food and Drug Administration (FDA) (42, 43). IRDye800cw, as a marker of SHRmAb antibodies, has been widely used because it has no obvious clinical toxicity evaluated in human trials (44). Therefore, it provides the possibility of improving the clinical translation of the Po-12 probe. Second, although peptides show superior performance in tumor diagnostic applications, their binding affinity is not yet comparable to that of specific antibodies. At present, the antibody-drug conjugate (ADC) has been used in tumor therapy as a very promising antitumor drug because of its high affinity and targeting (45). Therefore, using TIGIT as a naked antibody of ADC and further conjugation of NIR-II dye with peptide can not only further solve the limitations of this study but also further improve the efficacy of HCC immunotherapy. Finally, in contrast to bioluminescence imaging, which is affected by tissue depth and imaging dimension, PET/SPE-CT technology for small animals can achieve absolute quantification owing to the excellent penetration ability of radionuclides, with no signal attenuation; this provides three-dimensional information and accurate localization (46). Therefore, we need to construct PET imaging probes to evaluate tumor immune molecules in future studies.

## 5 Conclusion

In this study, we synthesized a TIGIT targeting NIRF probe, Po-12-Cy5. *In vitro* and *in vivo* experiments showed that Po-12-Cy5 was specifically absorbed by infiltrating lymphocytes in HCC. In addition, the probe could indicate TIGIT regulation of ^125^I seed radiation under NIRF guidance. As the TIGIT protein correlates with the degree of tumor differentiation and can be downregulated by RT, we believe that this probe can help indicate the regulation of the immune microenvironment of HCC by RT. Therefore, the Po-12-Cy5 probe can be used as an effective immunoevaluation tool with clinical translational potential.

## Data availability statement

The original contributions presented in the study are included in the article/**Supplementary Material**. Further inquiries can be directed to the corresponding author.

## Ethics statement

The studies involving human participants were reviewed and approved by IEC for Clinical Research of Zhongda Hospital, Affiliated to Southeast University. The patients/participants provided their written informed consent to participate in this study. The animal study was reviewed and approved by Animal Experimental Ethical Inspection Form of Southeast University.

## Author contributions

PZ, DS, and WS designed the experiments and carried out data analyses. SM, XY, and MS collated the data and performed the statistical analysis. YW and RC produced the initial draft of the manuscript. PZ finalized the manuscript and had final responsibility for the decision to submit for publication. All authors contributed to the article and approved the submitted version.
