# Supplementary material for: Identification of a novel peptide targeting TIGIT to evaluate immunomodulation of 125I seed brachytherapy in HCC by near-infrared fluorescence
Source: Front Oncol. 2023 Apr 14;13:1143266. doi: 10.3389/fonc.2023.1143266 (PMC10141647; doi:10.3389/fonc.2023.1143266)
Supplement: Supplementary file 1 [file DataSheet_1.docx]

Supplementary Material

**Identification of a novel peptide targeting TIGIT to evaluate** **immunomodulation of ^125^I seed brachytherapy in HCC by near-infrared fluorescence**

**Peng Zeng^1^*, Duo Shen^2^, Wenbin Shu^3^, Shudan Min^4^, Min Shu^4^, Xijuan Yao^5^, Yong Wang^5^, Rong Chen^1^**

*** Correspondence:** Peng Zeng: zpeng701l@163.com

**
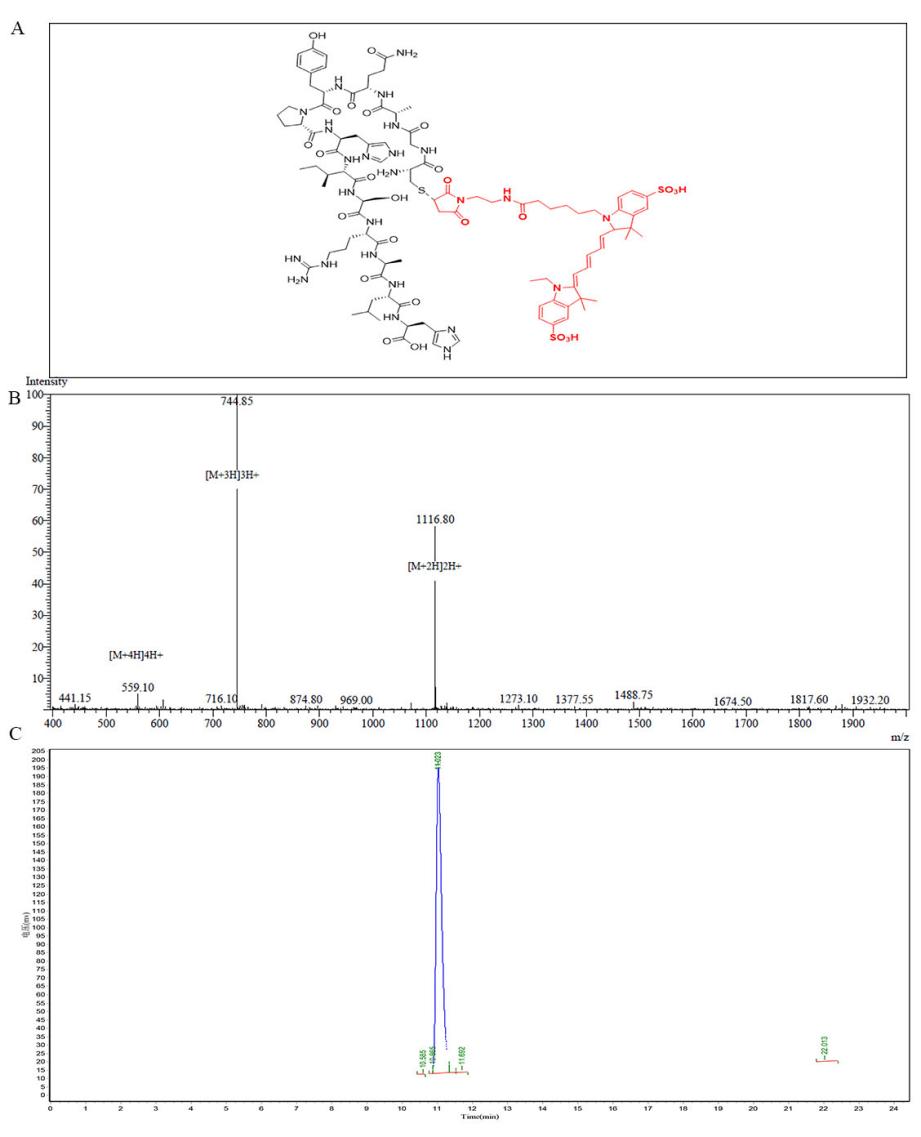
**

**Supplementary Figure 1.** The physicochemical characteristic of Po-12 peptide. (A) Chemical molecular structure of Po-12-Cy5; (B) Mass spectrometry profile of Po-12 peptide. (C) The stability of Po-12 peptide analyzed by analytical HPLC.

.
